# Synonymous site conservation in the HIV-1 genome

**DOI:** 10.1186/1471-2148-13-164

**Published:** 2013-08-04

**Authors:** Itay Mayrose, Adi Stern, Ela O Burdelova, Yosef Sabo, Nihay Laham-Karam, Rachel Zamostiano, Eran Bacharach, Tal Pupko

**Affiliations:** 1 Department of Molecular Biology and Ecology of Plants, Tel Aviv University, Tel-Aviv 69978, Israel; 2 Department of Cell Research and Immunology, Tel Aviv University, Tel-Aviv 69978, Israel; 3 Current address: Department of Microbiology and Immunology, University of California, San Francisco, USA; 4 Department of Integrative Biology, University of California, Berkeley, USA; 5 Current address: Department of Biotechnology and Molecular Medicine, University of Eastern Finland, Kuopio, Finland; 6 Current address: Center for Digestive Tract and Liver Diseases, Tel Aviv Sourasky Medical Center, Sackler Faculty of Medicine, Tel-Aviv University, Tel-Aviv, Israel; 7 Current address: Department of Biochemistry and Molecular Biophysics, College of Physicians and Surgeons, Columbia University, New York, NY 10032, USA

**Keywords:** Codon models, HIV-1, Synonymous substitutions, Silent substitutions, Ka/Ks

## Abstract

**Background:**

Synonymous or silent mutations are usually thought to evolve neutrally. However, accumulating recent evidence has demonstrated that silent mutations may destabilize RNA structures or disrupt *cis* regulatory motifs superimposed on coding sequences. Such observations suggest the existence of stretches of codon sites that are evolutionary conserved at both DNA-RNA and protein levels. Such stretches may point to functionally important regions within protein coding sequences not necessarily reflecting functional constraints on the amino-acid sequence. The HIV-1 genome is highly compact, and often harbors overlapping functional elements at the protein, RNA, and DNA levels. This superimposition of functions leads to complex selective forces acting on all levels of the genome and proteome. Considering the constraints on HIV-1 to maintain such a highly compact genome, we hypothesized that stretches of synonymous conservation would be common within its genome.

**Results:**

We used a combined computational-experimental approach to detect and characterize regions exhibiting strong purifying selection against synonymous substitutions along the HIV-1 genome. Our methodology is based on advanced probabilistic evolutionary models that explicitly account for synonymous rate variation among sites and rate dependencies among adjacent sites. These models are combined with a randomization procedure to automatically identify the most statistically significant regions of conserved synonymous sites along the genome. Using this procedure we identified 21 conserved regions. Twelve of these are mapped to regions within overlapping genes, seven correlate with known functional elements, while the functions of the remaining four are yet unknown. Among these four regions, we chose the one that deviates most from synonymous rate homogeneity for in-depth computational and experimental characterization. In our assays aiming to quantify viral fitness in both early and late stages of the replication cycle, no differences were observed between the mutated and the wild type virus following the introduction of synonymous mutations.

**Conclusions:**

The contradiction between the inferred purifying selective forces and the lack of effect of these mutations on viral replication may be explained by the fact that the phenotype was measured in single-cycle infection assays in cell culture. Such a system does not account for the complexity of HIV-1 infections *in vivo*, which involves multiple infection cycles and interaction with the host immune system.

## Background

Molecular evolutionary models are often applied to detect and quantify the type and the intensity of selective pressures acting on genes and on specific sites within them. Sites playing an essential role, such as those composing the active site of an enzyme, are unlikely to undergo amino-acid replacements over evolutionary time. This constraint reflects a purifying selection force, which prevents the fixation of mutations that otherwise would disturb protein structure or function [1,2]. The higher the intensity of purifying selection acting on a specific nucleotide or amino-acid site, the more conserved the site is expected to be. Hence, under the neutral theory of molecular evolution, conserved sites are assumed to indicate functional or structural importance, while variable sites may be regarded as evolving free from, or under weak, selective constraints. Some sites, however, are very fast evolving, yet are of great functional significance. Such fast evolution may reflect positive Darwinian selection. For example, the Influenza virus Hemagglutinin (HA) gene encodes a major surface antigen, targeted by neutralizing antibodies during infection. Sequence analysis of this gene suggested a few sites to be positively selected [3], and these were shown to be responsible for evading the host immune system. Taken together, sites that are under strong purifying or positive selection are likely to be functionally or structurally important and their identification is often a critical first step towards understanding and manipulating a protein’s function [Reviewed in [4,5].

In protein-coding genes, the selection type and intensity are usually inferred by contrasting the rate of nonsynonymous (amino-acid altering; Ka) to the rate of synonymous (silent; Ks) nucleotide substitutions [5]. The most currently-used models assume that the selective forces acting on protein-coding genes operate at the protein level only, while synonymous substitutions are free from selection and reflect the neutral rate of evolution [3]. In such a case, Ks is constant across codon positions, Ka is heterogeneous, and the inference of site-specific Ka/Ks values is based solely on Ka variation. Codon sites showing a Ka/Ks ratio significantly lower than 1 are regarded as undergoing purifying selection, suggesting that they are functionally or structurally important. Codon sites with Ka/Ks ratios significantly higher than 1 are indicative of positive Darwinian selection, suggesting adaptive evolution. Finally, sites inferred to evolve under a Ka/Ks ratio not significantly different from 1 are regarded as free from selection at the protein level [6]. A large number of studies have used this methodology to infer adaptive evolution in Human Immunodeficiency Virus type 1 (HIV-1), resulting from immune pressure or drug treatment [e.g., [7-9].

The assumption that Ks reflects the neutral rate of evolution suggests that synonymous substitutions should be fixed at a constant rate along the sequence. However, it has been shown that selection regularly operates on synonymous sites in different organisms [e.g., [10,11]. For example, purifying selection on synonymous sites was found in 9.4% of all yeast genes and 5.1% in all worm genes using a likelihood based model that distinguishes between synonymous substitutions between preferred and unpreferred codons [12]. Purifying selection on synonymous sites was suggested to result from considerations such as mRNA stability [13-17], splicing regulatory elements [e.g., [18], cis regulatory elements, and overlapping genes [11,19,20]. Furthermore, in bacteria and yeast, codon bias in highly expressed genes is the most documented type of selection on synonymous sites [e.g., [21]. Additionally, the choice among synonymous codons was shown to affect protein structure [22]. In addition, conservation of “optimal” and “non-optimal” codons was shown to reflect selection for cotranslational folding in yeast. Thus, it is becoming recognized that the evolutionary selective forces acting on synonymous sites reflect various biological phenomena [recently reviewed in [23].

The “Ks equal neutrality” assumption is particularly problematic in viruses since, due to their compact genome, regulatory elements are abundant within their protein coding regions. For example, there are well known RNA secondary structures along the HIV-1 genome, such as the Rev-Response Element (RRE), which plays important functional roles during the virus life cycle [24]. Another obvious example is the existence of overlapping genes, in which strong selection against both synonymous and nonsynonymous substitutions is expected. However, since the commonly used codon models fail to account for Ks variation, such regions are usually excluded from the analysis, despite their high biological interest. For example, to avoid overlapping regions, Yang et al. [9] excluded the entire *tat*, *rev*, and *vpu* genes and analyzed the evolutionary selective forces in the rest of the HIV-1 genome. Thus, ignoring Ks variability across sites is biologically unrealistic, problematic and may bias inference of selective forces across protein coding genes [25]. Furthermore, the ability to correctly model Ks variability across sites may reveal novel insights into the functional constraints acting on the DNA and RNA levels, which cannot be inferred using standard codon models.

Ngandu et al. [26] have previously applied a sliding-window approach to detect consecutive codons with low synonymous substitution rates within the HIV-1 genome. One of the regions discovered in that study was a 12-base-pair long region that resides within *env* for which no functional role had been previously described. Mutagenesis experiments in which synonymous mutations were introduced in this region, led to no observed difference between wild type and mutated clones in cell lines. Kosokovski-Pond et al. [27] have also applied models allowing for Ks variation to HIV-1 and other RNA viruses. However, their goal was to find shared evolutionary rate patterns across alignments rather than to detect conserved Ks regions.

In this study we constructed a genome-wide map of the selective forces operating against the fixation of synonymous substitutions across the HIV-1 genome. Our method relies on a model that explicitly accounts for dependency of synonymous rates among adjacent sites, thus removing stochastic noise from site specific Ks estimates [28]. Specifically, we developed the StrechFinder methodology that detects statistically significant consecutive synonymous-conserved sites. This methodology overcomes the inherent statistical problematic aspects of sliding window approaches for detecting evolutionary rate variation among sites [29]. We detected twenty-one short linear stretches within the genome, which display significantly lower levels of Ks than expected under neutrality. The forces leading to lowered Ks in these regions were explored. The majority of the identified regions were mapped to previously known regulatory functional regions, or to overlapping open reading frames (ORFs), validating our methodology as a general tool for predicting functional regions at the nucleotide level nested within protein-coding regions. Furthermore, we detected a number of Ks conserved regions that could not be assigned to a known function, and are thus predicted to be under selection due to a yet unknown reason. One such region is located within the *pol* gene. Mutagenesis experiments of this region were conducted to reveal its function but showed no clear effect on viral replication in cell culture. The implications of these results are discussed.

## Results and discussion

### Selection intensity on synonymous sites varies within and across HIV ORFs

A large dataset of 122 HIV-1 sequences spanning eight subtypes showed that variability in the synonymous substitution rate is present in each of the nine HIV-1 ORFs. The two evolutionary codon models that account for Ks variability (KaV-KsV and KaVD-KsD; see Methods) exhibited a significantly better fit to the data, compared to the commonly used KaV-KsC model that assumes a single Ks rate for all sequence sites (all *p*-values < 10^-34^; Table 1). Furthermore, for all ORFs other than *vpr*, significant support was found for the model accounting for dependency in evolutionary rates between adjacent sequence sites. The KaD-KsD model differs from the KaV-KsC model in two attributes: the dependence between adjacent sites and the variability in Ks. By comparing the differences between KaV-KsC and KaV-KsV and between KaD-KsD and KaV-KsV (Table 1), it is clear that assuming Ks variability leads to the dominant factor in the improved fit of the KaD-KsD model to the HIV-1 sequences.

**Table 1:** Maximum log-likelihood (LL) values for the nine HIV-1 ORFs under the KaD-KsD, KaV-KsV, and KaV-KsC models

**ORF**	**KaV-KsC**	**KaV-KsV**	**KaD-KsD**	***p*-value**^**a**^	***p*-value**^**b**^
Env	−95,879.1	−94,199.7	−93,958.9	< 10^-250^	< 10^-104^
Gag	−35,193.8	−34,706.5	−34,663.4	< 10^-230^	< 10^-18^
Nef	−21,444.5	−21,171	−21,114.8	< 10^-144^	< 10^-24^
Pol	−54,094.5	−53,342.7	−53,204	< 10^-250^	< 10^-60^
Rev	−10,892.2	−10,642.9	−10,639	< 10^-110^	0.02
Tat	−9,316.45	−9,103.74	−9,094.84	< 10^-97^	< 10^-3^
Vif	−13,368.2	−13,151.4	−13,141	< 10^-100^	< 10^-4^
Vpr	−7,158.27	−7,084.55	−7,082.61	< 10^-34^	0.14
Vpu	−10,188.1	−9,998.8	−9,974.56	< 10^-94^	< 10^-10^

^a^ Comparison of the KaD-KsD versus the KaV-KsC model.

^b^ Comparison of the KaD-KsD versus the KaV-KsV model.

**Figure 1:**
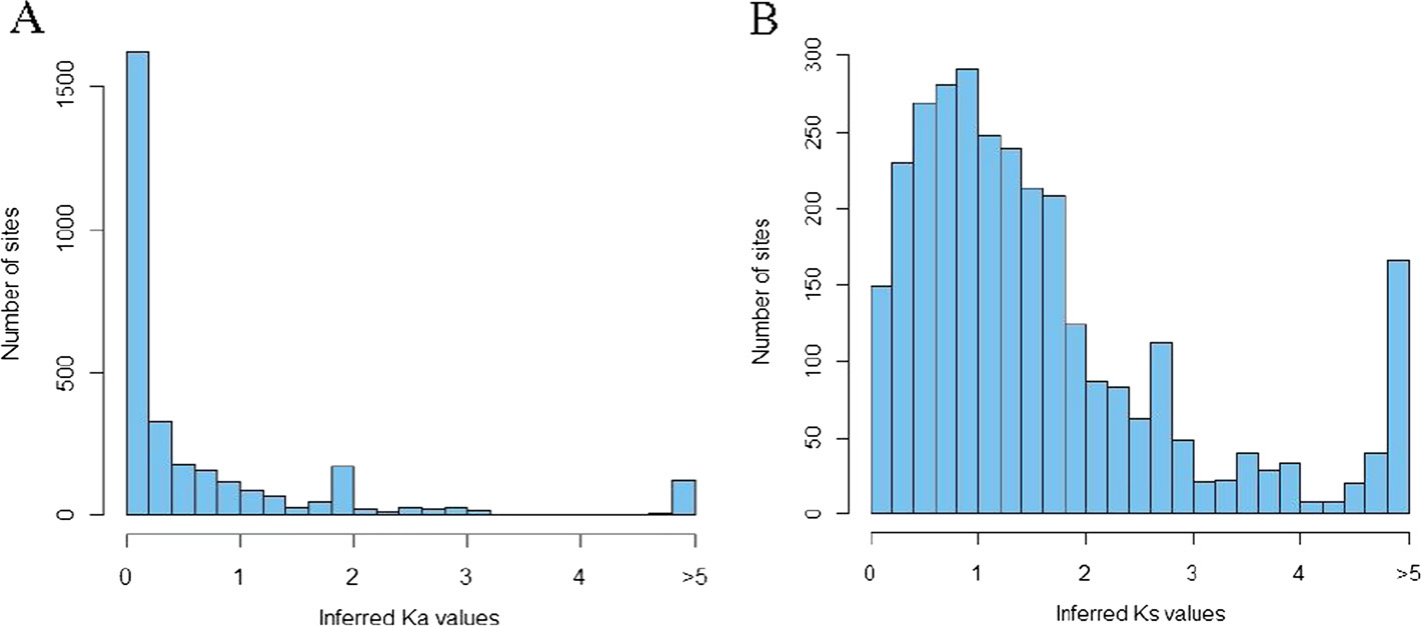
A histogram portraying the HIV-1 proteome-wide distribution of inferred (A) Ka and (B) Ks values.

Having established that KaD-KsD is the best fitting model, it was next used to infer site-specific Ka and Ks scores for each site in each of the ORFs. The posterior distributions of inferred Ka and Ks values were then examined (Figure 1). There is a large difference between the two distributions: On the one hand, most of the Ka distribution surrounds very low values, with an average of 0.56, supporting the notion that extensive purifying selection is acting on the coding sequences. On the other hand, the mass of the Ks distribution is centered around 1 (average 1.39), suggesting that most synonymous sites are under neutral selection. Indeed, the coefficient of variation (CV) of the Ka distribution is much higher than that of the Ks distribution (1.18 versus 0.71) suggesting higher level of variation of Ka compared to Ks values.

### Identification of Ks-conserved stretches across the HIV-1 genome

The StretchFinder algorithm (see Methods) was used to scan the estimated Ks values for extended regions exhibiting a statistically significant reduction of Ks. A stretch was defined as a consecutive region spanning between 2 and 40 codons. Assigning a *p*-value allowed the distinction between a region with a few randomly dispersed Ks-conserved sites and an extended region with low Ks values, which may be indicative of a functionally important region. This analysis yielded 21 stretches with significantly low values of Ks, following correction for multiple testing (Table 2). Notably, these stretches were very similar to those obtained when StretchFinder was applied to site specific Ks rates inferred using the KaV-KsV model. However, in a few cases the stretches obtained by the model assuming site dependency were slightly longer (results not shown). This can be explained by the smoothing effect induced by models assuming site dependencies.

**Table 2:** Summary of stretches with significantly low Ks, ranked according to StretchFinder

**Rank**	**ORF**	**Pos. in ORF**	**Overlap**	**Regulatory element**	**Remark**
1	*pol*	898-937		+	Contains the cPPT, the CTS and the DNA flap regions. The first two regions are involved in regulation of reverse transcription of the genome, and the third in translocation of the genome to the nucleus.
2	*vif*	173-186	+		Overlaps Vpr 1–14. In this region of Vpr there is a conserved homo-ologimerization domain.
3	*tat*	65-98	+		Overlaps Rev 18–52 (as well as Env 719–744), which includes the conserved homo-multimerization region and RNA-binding domain.
4	*pol*	986-1003	+		Overlaps Vif 1-18
5	*nef*	88-99		+	Contains the PPT (a primer for reverse transcription)
6	*tat*	41-52	+		Sites 47–52 overlap Rev 1–6.
					**Unexplained low Ks in sites 41-46**
7	*env*	728-744	+		Equivalent to region (3) (overlap of functional elements in Rev)
8	*pol*	7-31	+		Overlaps Gag 442–448 and Gag 454-463
9	*gag*	488-496	+		Overlaps Protease 1-9
10	*vif*	1-21	+		Equivalent to region (4) (overlaps Pol 986–1003)
11	*rev*	2-9	+		Overlaps Tat 49–56: conserved nuclear localization signal and RNA binding domain
12	*env*	535-541		+	RRE
13	*env*	2-5	+		Overlaps Vpu 56-60
14	*pol*	82-107			**Unexplained low Ks**
15	*gag*	2-5		+	Four RNA loops (SL1-4) of the HIV-1 packaging signal. Gag 2–5 overlaps the fourth loop SL4.
16	*env*	570-608		+	RRE
17	*vpu*	74-80	+		Overlaps Env.
18	*rev*	58-75	+		Overlaps Env 240-257
19	*pol*	939-954		+	Pol 939–947 forms the CTS end.
					**Unexplained low Ks at sites 948-954**
20	*env*	495-531		+	RRE
21	*vif*	114-143			**Unexplained low Ks**

Regions which could not be fully accounted for (whether as part of a regulatory domain or as part of an overlapped region) are marked in bold.

Seven of the conserved stretches were mapped to regions previously reported to have a functional role. The most statistically significant Ks conserved stretch along the HIV-1 genome region is located inside the *pol* ORF and spans positions 898 to 937. This region spans a few functional nucleic acid elements: the central polypurine tract (cPPT) [30], the central termination sequence (CTS), and the DNA flap region [31]. This stretch resides near a second significant stretch within *pol*, which spans positions 939 to 954 (Stretch 19 in Table 2). The conservation in this stretch may be partially explained by the presence of the end of the CTS within it. Indeed, this region within *pol* was previously identified using a manual scan for Ks conserved regions [28]. The second polypurine tract (PPT) in HIV-1 is located inside the *nef* gene and is also part of a stretch with significantly low Ks values (Stretch 5 in Table 2). A third known functional element that was detected as a significantly conserved stretch is SL4 [32,33], which serves as part of the RNA packaging signal and is located in *gag* (Stretch 15 in Table 2). Three out of five conserved stretches found in *env* (Stretches 12, 16, and 20) are mapped to the RRE region [34,35]. Additional twelve significantly conserved stretches are mapped to regions of overlapping genes. Indeed, in almost all of these cases the overlapped region was frame-shifted from the first stretch, and the low Ks values could be explained by selective constraints on the overlapped gene, i.e. by low Ka values in the other gene.

**Figure 2:**
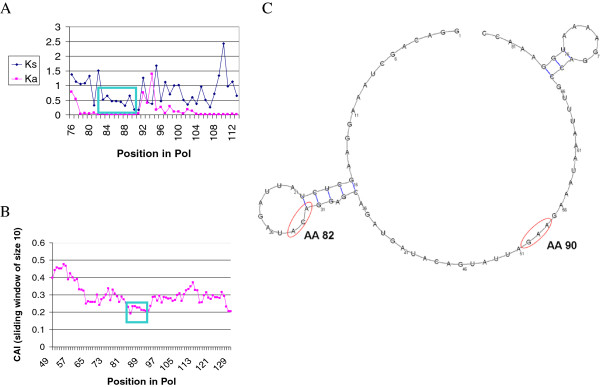
**Analysis of the *pol *82–90 Ks-conserved region. (A)** Ka and Ks values for each position in the protein. The *pol* 82–90 region is boxed. **(B)** Codon adaptation index analysis based on the human codon usage in highly expressed genes (see Methods). The *pol* 82–90 region is boxed. **(C)** RNA secondary structure prediction, including the flanking region (see text for details). Red ovals encircling triplets of nucleotides mark the beginning and end of *pol* 82–90 within the predicted region.

Of the 21 stretches in Table 2, the conservation of four stretches or regions within stretches could not be explained by a known regulatory domain or because of an overlap with other protein coding sequence. Among these four regions, we chose the stretch showing the most significant deviation from Ks homogeneity for further characterization. This stretch encompasses positions 82–107 within *pol*. Visual examination of the Ka and Ks values in this stretch (Figure 2A) revealed that the stretch is composed of two regions of decreased Ks, separated by a short peak. This led us to focus on the first region, spanning positions 82–90 of *pol*. We first tested whether the significant Ks conservation signal may be explained by codon bias, possibly affecting translation efficiency. The result of this analysis showed a slight decrease in the codon usage values in the *pol* 82–90 region (Figure 2B). Such a reduction is expected if indeed there are selective forces at the DNA/RNA level, as such forces do not allow substitutions toward optimal codons. As it has been shown that suboptimal codon usage in HIV is usually uncorrelated with translational efficiency [36], the reason for the conservation remains unknown.

We next tested whether secondary structure motifs within this stretch may explain the apparent Ks conservation. To determine the possible functional role of this region, an RNA secondary structure prediction was performed, and was compared to the experimental values obtained for the whole HIV-1 genome [37]. The predicted RNA secondary structure (Figure 2C) suggested that the *pol* 82–90 region is mostly unstructured, apart from a short stem and loop at the beginning of the stretch. This prediction matches the secondary structure model based on a high-throughput RNA analysis performed on the entire RNA genome of HIV-1 [37].

Taken together, these analyses reinforce the hypothesis that the low Ks of the *pol* 82–90 stretch is not related to its participation in RNA secondary structures and is most likely also unrelated to translational efficiency; thus, it may have a regulatory role, such as binding an unknown factor.

### Experimental testing

**Figure 3:**
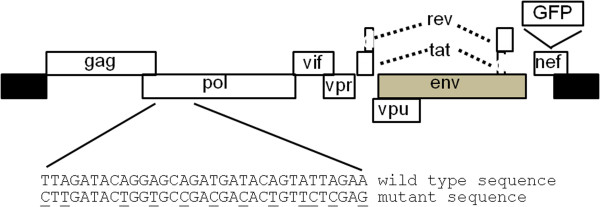
**Schematic presentation of the NLR+GFP clone with wild type and mutated sequence in the *pol *gene.** Shown are the HIV-1 reading frames. Black and gray rectangles represent the long terminal repeats (LTRs) and the out-of-frame *env* gene, respectively. GFP represents the GFP-expression cassette under the control of an internal CMV promoter. The mutated nucleotides are underlined. The indicated mutations do not change the amino acid sequence of the encoded protein.

We next proceeded towards revealing the biological role of the above conserved stretch comprising codon positions 82–90 of *pol* by mutagenesis experiments in molecular clones of HIV-1. We hypothesized that if these synonymous sites are indeed under selective constraints, artificially mutating these sites would affect the HIV-1 replication cycle. To this end, we introduced multiple synonymous mutations into the above *pol* region in the context of HIV-1 molecular clone NLR+GFP, generating the mutated NLR+GFP*pol*^mut^ clone (Figure 3 and Methods). Both the wild type and the mutated clones contain the entire HIV-1 genome sequence except the *env* and *nef* genes. In addition, the clones encode for the green fluorescent protein (GFP). We then used these clones to test for possible effects of the introduced synonymous mutations on both early (infectivity) and late (production) stages of the HIV-1 replication cycle, in human embryonic kidney 293T cells (HEK293T). Specifically, we aimed to determine whether these mutations affect early and/or late events of the virus replication cycle. Early events include virus entry into the host cells, reverse transcription, and integration of the HIV-1 genome into the host chromosomes. Late events include expression of the HIV-1 genes, assembly of the virion, its release from the infected cell, and maturation into fully infectious particles.

Disruption of early stages should result in decreased levels of integrated HIV-1 proviruses. To detect such a reduction, we measured the overall expression of genes (Gag and GFP) transcribed from the HIV-1 provirus. Searching for a possible effect of the mutations on late events, we quantified virion release from the cells by measuring capsid levels in the context of assembled virions. In addition, the infectivity of produced virions was monitored using single cycle infection assays.

**Figure 4:**
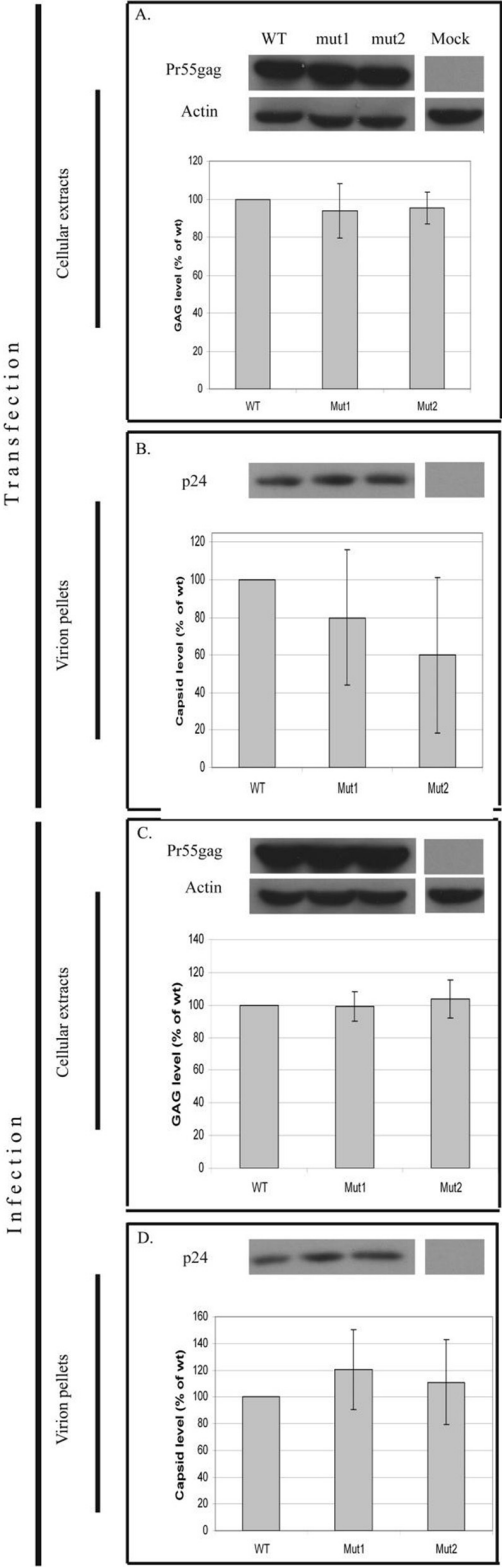
**Gag expression and virion release of the NLR+GFP and NLR+GFP*pol***^**mut **^**clones in transfected and infected cells. (A)** HEK293T cells were transfected with plasmids expressing the VSV-G envelope, and NLR+GFP (WT) or two identical clones of NLR+GFPpol^mut^ (mut1, mut2). Mock represents control cells transfected with no plasmid DNA. Gag precursor (Pr55gag) was detected in extracts of transfected cells (two days post transfection) by Western blotting using anti-capsid monoclonal antibody. Actin was used to control for protein levels in the samples. **(B)** Virions were purified from equal volumes of supernatants of cells in **(A)** and their levels were determined by detecting the capsid protein (p24), using Western blotting as above. **(C)** Equal amounts of virions from **(B)**, normalized by RT activity, were used to infect naïve 293T cells and the newly infected cells were analyzed two days post infection as in **(A)**. **(D)** Virions from supernatants of the cells indicated in **(C)** were analyzed as in **(B)**. For **(A-D)**, one representative Western blot is shown at the top of each panel and bars (bottom part) represent the average densitometry of the bands from three independent experiments.

Two identical (but separately prepared) clones of NLR+GFP*pol*^mut^ and the wild type clone NLR+GFP were transfected into HEK293T cells and their expression levels were examined by immunoblotting of the transfected cells extracts with a monoclonal antibody against HIV-1 capsid protein. This antibody detects both free capsid and Gag precursor proteins. Similar expression levels of the wild type and mutant clones were observed, as determined by quantifying the levels of the Gag precursor within transfected cells (Figure 4A). Thus, the mutations did not affect viral expression levels. Furthermore, applying the above analysis for the detection of virions purified from the supernatants of transfected cultures, yielded no significant difference between the production levels of HIV-1 particles of the tested clones (as deduced from measuring the levels of capsid in these preparations; Figure 4B). Overall, these analyses suggest that the mutations in *pol* did not hamper the late stages (expression, assembly and budding) of the HIV-1 replication cycle.

The above results cannot rule out possible effects of the mutations on viral infectivity. To further investigate this option, naïve HEK293T cells were infected with equal amounts of wild type and mutant virions, normalized by reverse transcriptase activity [as in [38]. To measure infection levels, infected cells were tested for either intracellular Gag or GFP levels, quantified by Western blot or flow cytometry analyses, respectively. Both Gag (Figure 4C) and GFP levels (not shown) were not significantly different among the wild type and mutated clones, suggesting that the mutations did not hamper infectivity. Finally, we tested whether HIV-1 particle production originating from infected cells (rather than transfected cells) varied between the tested clones, and here too, no significant difference was observed (Figure 4D). Overall, these results indicate that in the current experimental settings, the functional role of this conserved stretch has no (or only minimal) effect on the HIV-1 replication cycle. Possible reasons for the disagreement between the strong evolutionary conservation signal and the experimental results are discussed below.

## Conclusions

This study provides and explores a comprehensive map of the evolutionary selective forces operating on HIV-1. Previous studies inferring natural selection in HIV did not account for selection on synonymous substitutions, which may have possibly inflated estimates of positive selection [28]. Specifically, overlapping regions of genes are often considered problematic and are removed from analyses [9]. Here we show that complex inter-related selective forces may operate on all protein coding genes within HIV-1.

This analysis underscores the association between synonymously conserved regions and functional regions at the DNA or RNA levels. HIV-1 represents one of the most studied biological systems and thus provides us with the opportunity to correlate between the inferred Ks conserved stretches and known functional regions residing within coding regions. It is noteworthy that nearly all well studied regulatory regions residing within the ORFs of HIV-1 were also detected using our methodology. Nevertheless, four synonymously conserved regions were detected with no previously reported function. An important aspect of this work was the effort to experimentally elucidate the significance of the computational predictions derived from the evolutionary models. However, the mutagenesis assay performed here on one of the stretches (*pol* 82–90) resulted in insignificant differences between the wild type and mutated constructs. Insignificant differences between wild type and mutated clones were also obtained by Ngandu et al. [26], who experimentally tested the role for a synonymous conserved region within *env*. Notably, these experiments were performed in cell cultures, which do not necessarily mimic natural infections of CD4+ cells. For instance, interactions with salient host proteins (such as the interaction between Tetherin and Vpu) are often dependent on the specific cell-line used [39]. In addition, it is possible that these synonymous mutations reduce viral fitness, but this reduction is not significant enough to be revealed after only a single infection cycle. Finally, since this stretch may have an important role only in the context of the virus interaction with the host adaptive immune response, it was not discovered in the artificial cell culture experiments conducted here. Hence, future work is needed to determine the role of the novel conserved stretches detected here. Nevertheless, the methodology presented here is generic, and can be used to investigate other less-studied viral datasets to predict novel functional regions, as well as to explore the breadth of synonymous conservation in both cells and viruses. Moreover, the identification of conserved sites in viral populations should enhance PCR diagnostics of a variety of viral strains that otherwise significantly differ from each other, like in the case of HIV-1.

## Methods

### Evolutionary models

We have previously developed an evolutionary model that accounts for site variability and dependency among sites of both synonymous and nonsynonymous rates [28]. Using this model, Bayesian posterior estimates of both the Ka and Ks rates at each site can be computed. Briefly, this model specifies two rate distributions: the first accounts for the variability among synonymous sites and the second for the variability among nonsynonymous sites. In addition, the model explicitly accounts for dependencies between adjacent codon sites by incorporating two hidden Markov models (HMMs): the first representing dependencies between adjacent Ka rates and the second between adjacent Ks rates. Hereafter, this model is termed KaD-KsD. It was compared to two alternative models; the KaV-KsV model that allows for variation at both the Ka and Ks levels, yet assumes that adjacent sites evolve independently of each other, and the KaV-KsC model that assumes that Ks is constant across sites, Ka is variable across sites, and Ka values at adjacent sites are independent of each other. Since these are nested models, a likelihood ratio test (LRT) can be used to assess which model best fits the data. Specifically, differences in maximal log-likelihood (LL) values are compared to their expected values based on a chi-square distribution with 3 or 2 degrees of freedom when the KaD-KsD model is compared against the KaV-KsC or KaV-KsV models, respectively. If the best-fit model is either KaD-KsD or KaV-KsV, separate Ka and Ks values for each codon site can be estimated by calculating the expectation of the posterior rate distribution at each site. If the best-fit model is KaV-KsC, only Ka values for each site can be estimated.

### Identification of Ks-conserved stretches

Given the Ks estimates for each site, we aimed to obtain the set of linear stretches with significantly low Ks values. We thus developed the StretchFinder algorithm that tests for significant clustering of consecutive conserved codon sites. StretchFinder allows distinguishing between a region with a few randomly dispersed Ks-conserved sites and an extended region with low Ks values, which may be indicative of a functionally important region. Specifically, for a given sub-region of length *l*, the algorithm computes the average Ks value. Next, a null distribution of average Ks values is computed assuming no clustering of Ks conserved sites. Specifically, the null distribution is computed by shuffling the site specific Ks score across the entire alignment. The shuffling procedure is repeated *m* times and for each such shuffle, the average Ks score of a stretch of length *l* is computed. The set of average (shuffled) Ks values provides the null distribution. These null distributions were used to fit the parameters of an extreme value distribution (EVD). Finally a *p*-value for the score of a certain size was obtained from the corresponding EVD. In order to correct for multiple testing, the False Discovery Rate (FDR) method (Benjamini and Hochberg, 1995) was applied to correct each *p*-value. In this study we considered *l* =2 to 40 codons, and *m* =10^6^.

### Dataset

HIV-1 accession numbers were retrieved from the whole genome DNA multiple sequence alignment (MSA) available at Los Alamos HIV sequence database (http://www.hiv.lanl.gov/). Sequences were additionally filtered if: (1) not all nine ORFs were present in the corresponding GenBank file, (2) one of the coding sequences (CDS) was not divisible by three, (3) a stop codon was found in the middle of the CDS. This procedure resulted in 301 fully-sequenced genomes. The HXB2 sequence (accession number AF033819) was added to serve as a reference sequence in further analyses. Since the original Los Alamos MSA does not always maintain the reading frame, codon-based MSAs were created for each gene based on the translated protein MSAs created using the MUSCLE program [40]. In order to eliminate the number of gaps in the alignment, sequences that opened insertion positions shared by less than 2% of the sequences were removed, resulting in 122 HIV-1 aligned sequences. A whole proteome MSA was then generated by concatenating the nine MSAs generated for each protein separately. Maximum likelihood (ML) phylogeny reconstruction was performed based on the whole proteome alignment with the PhyML program [41], using among-site rate variation of four discrete rate categories, and the rtREV amino acid substitution matrix. Given the phylogenetic tree, the parameters of the three evolutionary codon models (KaV-KsC, KaV-KsV, and KaD-KsD) were optimized by ML for the whole-genome MSA as well as for each of the nine ORFs separately. Ka and Ks rates for each site were inferred over the whole proteome MSA, allowing comparison of the different values.

### Codon bias

The codon adaptation index (CAI) of a codon position in a sequence is defined as the ratio of the usage of the codon, to the usage of the most abundant codon for the same amino acid [42]. The usage of a codon is defined as the relative frequency of the codon in a dataset of highly expressed genes. The CAI of a sequence is defined as the geometrical mean of the values across the sequence. Here, since a multiple sequence alignment rather than a single sequence exists, the arithmetic mean (ω) of each codon position was calculated. The usage values of codons were taken from Haas et al. [43] based on a dataset of highly expressed human genes. To determine whether a stretch had deviant codon bias, CAI values were calculated for sliding windows of size 10 across the sequence, and plotted along the sequence. The stretch was manually inspected to see whether it was part of a peak or a depression as compared to the rest of the sequence.

### RNA secondary structure prediction

RNA secondary structure predictions were performed with the RNAalifold software [44]. The underlying method in this program takes into account both thermodynamic stability and sequence covariation, i.e., a stem structure is inferred only if supported by compensatory mutations along the alignment. The predictions were performed on regions spanning 30 nucleotides upstream and downstream of each stretch.

### Experimental methods

We cloned an internal GFP expression cassette into the Nef ORF of the NLR+HSA construct, which is a derivative of the HIV-AP clone [45], to create the NLR+GFP plasmid (Figure 3). The NLR+GFP vector contains the entire HIV-1 genome sequence and expresses all viral proteins except Env and Nef. This construct allows us to compare infectivity (provided that envelope proteins were supplemented) of the NLR+GFP clone versus a clone (NLR+GFP*pol*^mut^), in which twelve synonymous mutations were introduced into this stretch. Notably, these 12 silent point mutations in the 33 base long sequence were chosen to maximize the number of synonymous changes without altering the protein’s amino-acid sequence. When applicable, double synonymous mutations per codon were introduced (e.g., TTA to CTC); otherwise, transversions were preferred over transitions, and the resulting codon was chosen as the one with the lowest codon usage in HIV, according to [46]. In addition, the introduction of this vector into the host cells resulted in the expression of a cloned GFP, thus allowing easy monitoring of the viral RNA expression by fluorescent microscopy or flow cytometry analysis. HEK293T cells (approximately 80% confluency in 60 mm plates) were transfected with a mixture of plasmids consisting of 10 μg of NLR+GFP (or NLR+GFP*pol*^mut^) clone and 2.5 μg of pMD.G (expressing the vesicular stomatitis virus envelope glycoprotein) by the calcium phosphate method. Two days post transfection, virions were purified from supernatants (3 ml) of transfected cultures by ultracentrifugation through a 25% sucrose cushion and the transfected cells were harvested and extracted. Gag and Capsid proteins were detected in extracts of transfected or infected (two days post infection) cells by immunoblotting using the monoclonal antibody against HIV-1 capsid protein (from hybridoma clone 183-H12-5C; NIH AIDS Research and Reference Program). Exogenous reverse transcriptase (RT) assay was performed to quantify HIV particle levels in supernatants of transfected cells. These supernatants, normalized to have equal amounts of virions, were diluted, supplemented with 8 μg/ml polybrene and used to infect naive HEK293T cells. Two days post infection, a portion of infected cells was extracted for immunoblotting and a portion was analyzed for GFP fluorescence, using flow cytometry. All the above methods were conducted as described previously [38,47,48].

## Competing interests

The authors declare that they have no competing interests.

## Authors’ contributions

IM, AS, EB and TP conceived of the study. IM and AS compiled the sequence data and performed the computational analyses, IM, EB, YS, NLK, RZ carried out the molecular analyses, IM, AS, EB and TP drafted the manuscript. All authors finalized and approved the manuscript.
